# Thiophenylazobenzene: An Alternative Photoisomerization Controlled by Lone‐Pair⋅⋅⋅π Interaction

**DOI:** 10.1002/anie.201909739

**Published:** 2019-11-18

**Authors:** Chavdar Slavov, Chong Yang, Andreas H. Heindl, Hermann A. Wegner, Andreas Dreuw, Josef Wachtveitl

**Affiliations:** ^1^ Institute of Physical and Theoretical Chemistry Goethe University Frankfurt am Main Germany; ^2^ Interdisciplinary Center for Scientific Computing (IWR) University of Heidelberg Heidelberg Germany; ^3^ Institute of Organic Chemistry Center for Materials Research (LaMa) Justus Liebig University Giessen Germany

**Keywords:** isomerization mechanisms, photochromism, photoswitches, thiophenylazobenzene, time-resolved spectroscopy

## Abstract

Azoheteroarene photoswitches have attracted attention due to their unique properties. We present the stationary photochromism and ultrafast photoisomerization mechanism of thiophenylazobenzene (TphAB). It demonstrates impressive fatigue resistance and photoisomerization efficiency, and shows favorably separated (*E*)‐ and (*Z*)‐isomer absorption bands, allowing for highly selective photoconversion. The (*Z*)‐isomer of TphAB adopts an unusual orthogonal geometry where the thiophenyl group is perfectly perpendicular to the phenyl group. This geometry is stabilized by a rare lone‐pair⋅⋅⋅π interaction between the S atom and the phenyl group. The photoisomerization of TphAB occurs on the sub‐ps to ps timescale and is governed by this interaction. Therefore, the adoption and disruption of the orthogonal geometry requires significant movement along the inversion reaction coordinates (CNN and NNC angles). Our results establish TphAB as an excellent photoswitch with versatile properties that expand the application possibilities of AB derivatives.

## Graphical abstract

**My own way**: Thiophenylazobenzene is a sulfur‐based azoheteroarene photoswitch with impressive fatigue resistance, photoisomerization efficiency, and highly selective photoconversion. Its (*Z*)‐isomer adopts an unusual orthogonal geometry stabilized by an intramolecular lone‐pair⋅⋅⋅π interaction governing the ultrafast photoisomerization, which requires a mechanism involving significant movement along the inversion reaction coordinate.

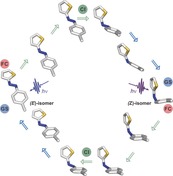

## Introduction

The operation of molecular photoswitches is based on the reversible transformation of the switching molecule between states of different physicochemical properties (for example, geometrical structure, dipole moment, absorption spectrum, or redox potential).^1^ The utilization of light as a trigger allows easy manipulation, which, combined with the instantaneous property change of the molecule, makes photoswitches extremely attractive for applications in chemical science and technology.^2^

Azobenzenes (AB) are a prominent group of widely utilized photoswitches. They are highly fatigue‐resistant and relatively easy to synthesize.^3^ The photochromism of ABs is based on the *E*⇄*Z* photoisomerization of the central N=N bond, which results in substantial geometric and spectral changes.^4^ Spectrally, ABs are characterized by a strong π→π* transition band (S_2_), typically located in the 300–350 nm range for the (*E*)‐isomer and the 250–290 nm range for the (*Z*)‐isomer. They also show a weakly allowed n→π* transition band (S_1_) in the 400–450 nm range for both isomers. The photoisomerization quantum yield (QY) of ABs is excitation‐wavelength‐dependent: QY_*E(*π→π*)→*Z*_ *≈13 %,* QY_*E(*n→π*)→*Z*_ *≈29 %,* QY_*Z(*π→π*)→*E*_ *≈24–36 %,* QY_*Z(*n→π*)→*E*_ *≈47–51 %*.^4^, ^5^

The *E*→*Z* photoisomerization of ABs proceeds on the sub‐ps to ps timescale after both n→π* and π→π* excitations. After S_2_ excitation, ultrafast (≈100 fs) relaxation to the S_1_ state is observed.^6^ The decay of the S_1_ state is described by two lifetimes (≈400 fs and ≈2 ps), originally attributed to direct and diffusive motion from the Franck–Condon region to the conical intersection with the ground state.6b, 6c, 7 Relaxation lifetimes after S_1_ excitation are slightly different, indicating that the relaxation pathways are differently populated.6b, 6d Recently, only the longer (≈2 ps) S_1_ decay component was associated with *E*→*Z* isomerization, while the shorter one (≈400 fs) was assigned to a nonreactive relaxation pathway from a region on the S_1_ potential energy surface (PES) that is accessible only after S_2_ excitation.6f While this result designates an important tendency, it does not yet explain the presence of a similar (≈400 fs) lifetime component after S_1_ excitation. Also, additional pathways may be involved, making a clear distinction between the timescales of the relaxation pathways in the ultrafast data hard.^8^ In fact, lifetime‐distribution analysis has shown that the two S_1_ relaxation lifetimes discussed above belong to a broad ≈700 fs lifetime distribution.6e This indicates a significant overlap of the timescales of the reactive and the nonreactive pathways.^8^ The *Z*→*E* photoisomerization is ultrafast with a main lifetime of ≈150 fs and a minor contribution of ≈1 ps lifetime.6b, 6d, 7a In both isomerization directions, relaxation to the ground state is followed by vibrational cooling (10–20 ps lifetime).6b, 6e, 6f, 7a, 9

The photoisomerization mechanism of ABs has been analyzed extensively by quantum‐chemical calculations.^4^ In the latest works, a consensus arises that inversion‐assisted rotation is the dominant photoisomerization mechanism, particularly in the condensed phase.6f, 8, 10 The lower isomerization QY after π→π* (S_2_) excitation was attributed to a nonreactive internal‐conversion channel from the S_1_ to the ground state accessible only after S_2_→S_1_ relaxation6f, 10f or to a nonreactive channel due to crossing of the S_2_ and S_3_ PESs.^11^

In contrast to the conventional, well‐studied ABs, the photochemical properties of azoheteroarenes remain largely unexplored. Recent reports reveal that azoheteroarenes, like arylazopyrazoles and arylazopyrroles, can adopt different ground‐state conformations stabilizing or destabilizing the (*Z*)‐isomer, which allows for a remarkable tunability of the thermal relaxation rate from seconds to ≈1000 days.^12^ In other azoheteroarenes, impressively high thermal‐relaxation rates on the microsecond^13^ and even the nanosecond^14^ timescale have been achieved. Furthermore, due to distortions because of steric effects, the spectral properties of azoheteroarenes are altered. This often results in a favorable separation of the (*E*)‐ and the (*Z*)‐isomer bands that permits >98 % conversion in each direction.12a, 12b, 12d Evidently, choice, position, and orientation of the heteroaryl and its substituents represent a new tuning dimension for ABs. Moreover, the presence of heteroatoms permits new functional designs previously unavailable in conventional ABs. Therefore, azoheteroarenes offer an untapped potential for further optimization and expansion of the capabilities of AB photoswitches.

In this work, we explore a different azoheteroarene design, where one of the phenyl groups of a conventional AB is substituted by a thiophenyl group. We present its synthesis along with the theoretical and experimental investigation of the photoisomerization of this thiophenylazobenzene (TphAB) photoswitch.

## Results and Discussion

### Synthesis

**Scheme 1:**
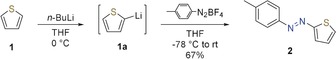
Synthesis of thiophenylazobenzene **2**.

The investigated TphAB **2** was synthesized based on the coupling of aryldiazonium salts with aryllithium compounds^15^ (Scheme 1). Herein, thiophene (**1**) was readily lithiated at the 2‐position and subsequently added to a 4‐methylphenyldiazonium tetrafluoroborate suspension at low temperature to obtain TphAB **2** in 67 % yield (see Supporting Information).

### Photochromic Properties

**Figure 1:**
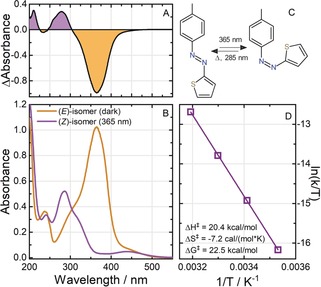
A) Photostationary state PSS_365_: (*E*)‐isomer absorbance‐difference spectra; B) Absorption spectra of the (*E*)‐isomer and the PSS_365_ (see Figure S5 for the pure (*Z*)‐isomer spectrum); C) TphAB isomerization; D) Eyring plot for the thermal *Z*→*E* relaxation (see also Table S1 for the used parameters).

All spectroscopic experiments with TphAB were performed in acetonitrile. The absorption spectrum of the thermodynamically stable (*E*)‐isomer of TphAB shows a dominant π→π* absorption band at 365 nm and a weaker n→π* band at ≈450 nm (Figure 1 A,B). Irradiation of the (*E*)‐isomer with 365 nm leads to *E*→*Z* photoisomerization of the central N=N bond (Figure 1 C). The (*Z*)‐isomer is characterized by two main absorption bands: i) a π→π* absorption band at ≈285 nm, which has about half the intensity of the (*E*)‐isomer π→π* band (365 nm); and ii) an n→π* band at ≈450 nm, which is only about as intense as the one of the (*E*)‐isomer. Due to the identical spectral location and intensity of the n→π* bands of the (*E*)‐ and the (*Z*)‐isomers, the *Z*⇄*E* photoconversion of TphAB is achieved by irradiation in the corresponding π→π* absorption bands.

The *E*→*Z* conversion under 365 nm irradiation is extremely efficient, resulting in less than 3 % (*E*)‐isomer in the photostationary state PSS_365_ (Figure S5, Supporting Information). This unusually high photoconversion level is due to the favorable separation of the (*E*)‐isomer π→π* band from the (*Z*)‐isomer absorption band in TphAB. The (*Z*)‐isomer content in PSS_285_ is also very low (≈13 %). The QY determination (see Supporting Information and ref. 16) for the two photoisomerization reactions of TphAB after π→π* excitation reveals some of the highest ever reported QYs for an AB system, with an impressive QY_*E*(π→π*)→*Z*_ of ≈44 % and QY_*Z*(n→π*)→*E*_ of ≈65 %. These QYs are even significantly higher than the ones typically reported for n→π* excitation of the conventional ABs.^4^ TphAB shows very high fatigue resistance to repeated photoswitching. After 50 photocycles equivalent to ≈9 h of high‐intensity light exposure, ≈3 % degradation at most is detected (Figure S1).

The *Z*→*E* thermal‐relaxation time of TphAB is considerably shorter than that of AB, with a half‐life of ≈120 min (20 °C). The temperature dependence of the thermal relaxation rate was determined at four different temperatures between 10 °C and 40 °C to obtain the thermodynamic parameters for the corresponding transition state (Figure 1 D).

### Quantum‐Chemical Calculations

**Figure 2:**
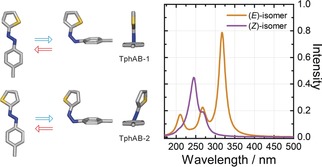
Left: Geometry‐optimized conformations adopted by TphAB. Right: absorption spectra calculated using the ADC(2) method (see Table S1 and Figures S3 and S4), taking into account the distribution of the conformations.

Theoretical calculations using the second‐order algebraic diagrammatic construction scheme for excitation energies (ADC(2))^17^ and linear‐response time‐dependent density functional theory (TDDFT;^18^ see Supporting Information) were performed to gain insight into the molecular properties of the studied TphAB. A ground‐state geometry optimization of TphAB yielded two stable geometries for both the (*E*)‐ and the (*Z*)‐isomer with different rotational orientations of the thiophenyl ring with respect to the azophenyl group—TphAB‐1 and TphAB‐2 (see Figure 2, left). Similar to AB, the (*E*)‐isomers of TphAB are planar. However, the (*Z*)‐isomers of TphAB adopt a rather unusual geometry where the thiophenyl ring lies in plane with the CNNC moiety, while the phenyl ring is either perfectly orthogonal to this plane (TphAB‐1) or slightly twisted away from it (TphAB‐2; Figure 2, left). This is in stark contrast to AB^4^ and the related azothiophene,^19^ where both rings are twisted away from the CNNC plane. Similar unusual geometries of the (*Z*)‐isomer have been reported for some nitrogen‐based azoheteroarenes,12a, 12b, 12d but not for the conventional AB. Interestingly, it was found that in these compounds, the orthogonal geometry is disfavored when a bulky substituent is present in *ortho* position to the CNNC group.12d Alternatively, the orthogonal geometry is adopted when an H‐atom is present in *ortho* position due to a favorable C−H⋅⋅⋅π interaction.12d Remarkably, TphAB adopts an orthogonal geometry only when the S‐atom, which is in *ortho* position, faces the phenyl ring (TphAB‐1, Figure 2 left), while the twisted geometry is realized when the S‐atom is facing away from the phenyl group (TphAB‐2, Figure 2 left). The differences in the conformations adopted by TphAB and by nitrogen‐based azoheteroarenes12d can be explained by differences in the attractive and repulsive interactions. In TphAB‐1, the orthogonal structure of the (*Z*)‐isomer is stabilized by a favorable interaction between the lone pair of the S‐atom and the π‐system of the phenyl ring (lone‐pair⋅⋅⋅π interaction^20^). Based on the results from azoheteroarenes,12d one would also expect the adoption of an orthogonal structure in the TphAB‐2 (*Z*)‐isomer, where the *ortho* H‐atom on the thiophenyl ring is involved in a C−H⋅⋅⋅π interaction. However, in this configuration (TphAB‐2), where the S‐atom is facing away from the phenyl ring, the lone pair of the S‐atom comes into close contact with the lone pair of the N‐atom of the azo group, which results in a repulsive interaction and the ensuing twisting of the thiophenyl ring.

Although the geometry optimization of TphAB yields two stable geometries, the twisted and the orthogonal conformation of the (*Z*)‐isomer, their Boltzmann distribution indicates that at room temperature, the orthogonal structure (TphAB‐1) represents ≈99.5 % of the population. Interestingly, the Boltzmann distribution of the (*E*)‐isomers also shows that the TphAB‐1 configuration is dominant (≈97.2 %). The excitation energies for all isomers obtained from the theoretical calculations (Figures S3 and S4, using the ADC(2) and BHLYP methods) and the Boltzmann distributions were used to simulate the theoretical absorption spectra of the isomer mixtures (Figure 2, right, and Figure S5). These calculated spectra reproduce the experimental spectra of the (*E*)‐ and (*Z*)‐isomers of TphAB in acetonitrile (Figure 1 B) very well given an energy blue‐shift of ≈0.61 eV. For the (*E*)‐isomer, a strong transition is present at ≈320 nm, which has π→π* character (see Figure S2 for attachment/detachment densities). The n→π* transition is only weakly allowed in the (*E*)‐isomer and therefore the intensity in the >400 nm range is small. The calculated spectrum of the (*Z*)‐isomer of TphAB shows a strong contribution in the 250–300 nm region due to two π→π* transitions (Figure 2, right). The attachment/detachment densities for the dominant TphAB‐1 configuration (Figure S2) show a very interesting character for these π→π* transitions: The lower‐energy transition is located entirely on the azothiophenyl group, while the higher‐energy transition shows charge‐transfer character from the phenyl to the thiophenyl group. Concerning the n→π* transition of the (*Z*)‐isomer, it appears that in the orthogonal geometry, the transition is very weak and has negligible contribution to the absorption spectrum (Figure 2, right) in contrast to AB. This result is in agreement with the experiential absorption spectrum (Figure 1 B), where the intensity in the >400 nm region is very similar for both the (*E*)‐ and the (*Z*)‐isomer. Noteworthy, in the twisted geometry of the (*Z*)‐isomer, the n→π* transition is stronger (Figure S3 and S4) due to the smaller angle between the plane of the nonbonding orbitals of the azo group and the azothiophenyl plane. Nevertheless, the contribution of the twisted geometry to the experimental spectrum is negligible as this configuration is essentially not present at room temperature (see above).

### Ultrafast E→Z Photoisomerization

**Figure 3:**
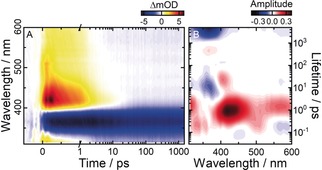
A) Transient‐absorption data of (*E*)‐TphAB measured in acetonitrile after 355 nm excitation (π→π* band): GSB, negative absorption difference signal, light to dark blue; excited‐state and hot‐ground‐state absorption, positive absorption difference signal, yellow to red to black. B) Corresponding LDMs obtained from the lifetime‐distribution analysis (see Supporting Information) of the time‐resolved data in (A). LDMs are read as decay‐associated spectra from global‐lifetime analysis: positive (red) amplitudes account for decay of absorption or rise of GSB; negative (blue) amplitudes account for rise of absorption or decay of GSB.

The *E*→*Z* photoisomerization of TphAB after 355 nm excitation in the π→π* absorption band of the (*E*)‐isomer was studied by ultrafast transient absorption spectroscopy (see Supporting Information and ref. 16 for a description of the pump–probe setup). The ultrafast dynamics (Figures 3 A and S6 A) resemble that of the conventional AB.6b, 6e However, the main excited‐state absorption (ESA) bands appear shifted to the red by ≈30 nm (to 510 nm and 420 nm). On the sub‐250 fs timescale, the ESA located at ≈510 nm decays concomitantly with the rise of the ESA at 420 nm (Figure S6). In turn, the decay of the 420 nm ESA proceeds on the early ps timescale but is not associated with a complete recovery of the ground‐state bleach (GSB) band at 365 nm. Instead, a hot ground‐state band contribution is observed on the lower‐energy side of the GSB, which decays on the 10 ps time scale. No product‐band formation is visible in the transient absorption data in the detected wavelength range. This is due to the dominant absorption of the (*E*)‐isomer in this spectral window, which obscures the formation of the (*Z*)‐isomer. Nevertheless, the formation of the (*Z*)‐isomer can be indirectly deduced by the strong nondecaying GSB signal on the nanosecond timescale, which is indicative for the depopulation of the initial (*E*)‐isomer.

The experimental data were analyzed by lifetime‐distribution analysis (see Supporting Information and ref. 21) and the corresponding lifetime‐distribution map is presented in Figure 3 B. The decay of the 510 nm ESA is described by a ≈100–200 fs lifetime distribution (positive amplitude) and can be assigned to the decay of the initially excited π→π* state (S_2_) of the (*E*)‐isomer into the n→π* state (S_1_; negative amplitudes). The lifetime distributions for this process are not fully resolved due to the limited time resolution (≈100 fs) of the experiments. Based on similar studies of the conventional AB with higher time resolution,6d, 6f it can be expected that the lifetime of this relaxation is even shorter. Therefore, in our experiments, this lifetime possibly overlaps with the lifetime describing the relaxation on the S_1_ surface. The decay of the 420 nm ESA, ascribed to the n→π* state (S_1_), is characterized by a relatively broad lifetime distribution centered at 950 fs (positive amplitude). A similar albeit slightly shorter lifetime distribution was found in AB.6e Therefore, the decay of TphAB from the excited state S_1_ to the ground state appears to be slightly slower. Typically, this decay dynamics is fitted by two lifetime components6b, 6f via conventional global lifetime analysis.^21^ However, lifetime‐distribution analysis indicates that those are artificially assigned to describe the rather broad distribution of relaxation processes. Nevertheless, it was proposed that the slower S_1_ relaxation pathways are reactive, while the fast ones are nonreactive.6f, 8 In this respect, the shift of the corresponding lifetime distribution in TphAB towards longer lifetimes (dominance of the reactive pathways) may potentially explain the much higher isomerization QY (44 %, see above) as compared to AB (≈10 %).^4^, ^5^

After the relaxation of the S_1_ state, a pair of a negative (365 nm) and a positive (405 nm) 10–20 ps elongated and tilted lifetime distributions are observed that describe the nonexponential cooling dynamics in the ground state.

### Ultrafast Z→E Photoisomerization

**Figure 4:**
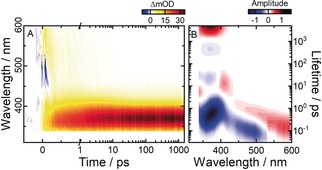
A) Transient‐absorption data of the (*Z*)‐TphAB measured in acetonitrile after 455 nm excitation (n→π* band). B) Corresponding LDMs obtained from the lifetime‐distribution analysis of the time‐resolved data in (A).

The ultrafast *Z*→*E* photoisomerization of TphAB was investigated after 455 nm excitation in the n→π* (S_1_) absorption band of the (*Z*)‐isomer. On the sub‐250 fs timescale, the transient‐absorption data (Figures 4 A and S6 B) shows a broad ESA signal over the complete detection range. This ESA is interrupted only by the GSB at ≈450 nm (in the range of the n→π* absorption of the (*Z*)‐isomer). This early ESA undergoes an ultrafast blue‐shift, which results in complete ESA/GSB signal compensation and an ESA rise in the 365 nm range. Therefore, after ≈200 fs, only positive transient absorption is present in the detected spectral range. The ESA then decays on the sub‐ps to the low ps timescale. This decay is most obvious above 430 nm, while below 430 nm, the ESA transforms into the product‐absorption signature (330–420 nm). The product absorption continues to grow strongly on the ps timescale and results in an intense long‐lived product band associated with the efficient *Z*→*E* isomerization. Initially, the product band appears broader on the red (≈400–450 nm) spectral side. However, this red side undergoes a blue‐shift (from ≈420 nm to 365 nm) on the 10 ps timescale due to the cooling of the hot ground‐state product (Figure 4 A).

The ultrafast dynamics of the (*Z*)‐isomer is exceptionally nonexponential, as illustrated by all lifetime distributions below 1–2 ps (Figure 4 B). Above 425 nm, a pair of a positive and negative, tilted and elongated lifetime distributions stretches from <100 fs to ≈1 ps. They account for the ultrafast, nonexponential excited‐state relaxation dynamics that proceeds from the S_1_ Franck–Condon region towards the S_1_ minimum and through the conical intersection with the ground state. Similarly, in the 350–430 nm range, a strong, elongated, and tilted (from short to long wavelengths) negative lifetime distribution is present that also stretches from <100 fs to 1–2 ps. The shorter, blue side of this distribution is associated with the early ESA‐shift dynamics due to the relaxation on the S_1_ PES, while the longer red side is associated with the S_1_ to S_0_ transition and the formation of the hot ground‐state product band. Again, due to the strong nonexponentiality of the dynamics, the relaxation on the S_1_ and to the ground state cannot be observed as separate lifetime distributions. The cooling dynamics of the hot ground‐state photoproduct is described by a pair of a positive and a negative distribution at ≈8–20 ps around 350–430 nm (Figure 4 B).

### Photoisomerization Mechanism

It is generally accepted that the torsion of the central CNNC moiety plays a dominant role in the isomerization of ABs.6f, 8, 10 Therefore, we performed relaxed PES scans along the torsion coordinate of TphAB in the S_1_ (n→π*) state (Figure S7). The resulting PESs for the orthogonal (TphAB‐1) and the twisted (TphAB‐2) geometries resemble the shape of those in AB, with a conical intersection with the ground state at a CNNC angle of ≈90°.

**Figure 5:**
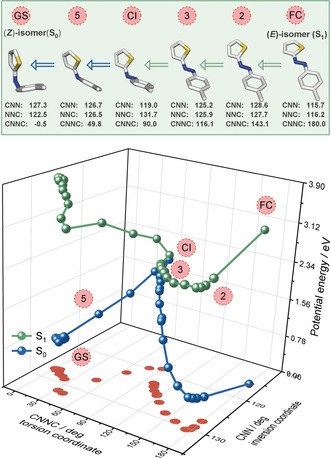
Top: Example geometries from the unconstrained/partially constrained (∡CNNC 116° to 90°) optimization of (*E*)‐TphAB‐1 illustrating the isomerization mechanism on the S_1_ PES. Bottom: PES obtained from the unconstrained/partially constrained optimization of (*E*)‐TphAB‐1 started in the Franck–Condon region on S_1_. Note: CNN is the angle on the thiophenyl side of the molecule, while NNC is the angle on the phenyl side.

**Figure 6:**
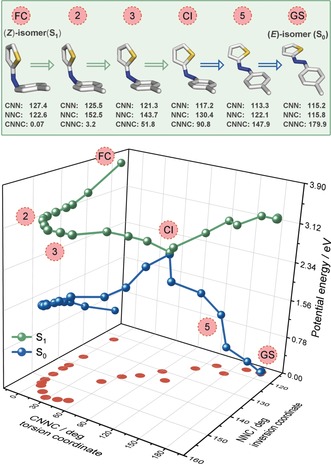
Top: Example geometries from the unconstrained optimization of (*Z*)‐TphAB‐1 illustrating the isomerization mechanism on the S_1_ PES. Bottom: PES obtained from the unconstrained optimization of (*Z*)‐TphAB‐1 started in the Franck–Condon region on S_1_. Note: CNN is the angle on the thiophenyl side of the molecule, while NNC is the angle on the phenyl side.

However, given the unconventional geometry adopted by the (*Z*)‐isomer (Figure 2), we decided to further investigate the contribution of other reactive coordinates to the isomerization mechanism operating in TphAB. In principle, nuclear‐dynamics simulations would be required to obtain a dynamical picture of the reaction mechanism, which are not feasible at present. Instead, we performed unconstrained geometry optimizations for both the (*E*)‐ and (*Z*)‐TphAB‐1 (orthogonal geometry) starting from the corresponding Franck–Condon geometries in S_1_ (see the Supporting Information for details). These calculations mimic the structural relaxation processes and identify the relevant reaction coordinates. The shape of the PESs obtained along theses optimizations allows for conclusions with respect to the dynamic mechanism. The optimizations bring the molecule from the Franck–Condon region to the conical intersection with the ground state. At this point, we switched the unconstrained optimization to the S_0_ PES to obtain the complete isomerization pathway. The optimizations of (*E*)‐ and (*Z*)‐TphAB‐1 unveil contributions of further coordinates beyond the typical CNNC torsion. For clarity, we name the angle on the thiophenyl side of the molecule CNN, while NNC corresponds to the angle on the phenyl side and examine the changes along these two coordinates (Figures 5, 6, S8 and S9; Tables S2 and S3).

### Isomerization Mechanism of (E)‐TphAB‐1

The unconstrained optimization of (*E*)‐TphAB‐1 in the S_1_ state shows a relatively large increase (≈15°) in both the CNN and the NNC angles, while the CNNC angle remains at ≈180° (Figures 5 and S8). This is followed by a large decrease of the CNNC angle to 116° and, simultaneously, a minor decrease in the CNN and the NNC angles to ≈125° (Figure 5, point 3, and Figure S8). At this point, the unconstrained optimization converges to a local minimum at a CNNC angle of 90° due to the presence of a small barrier on the S_1_ PES on the way to the conical intersection with the ground state. From this point, we performed constrained geometry optimizations with fixed CNNC angles between 116° and 90° to reach the conical intersection. During this torsion of the CNNC angle, the NNC angle increases back to ≈132°, while the CNN angle decreases further to ≈120°. At a CNNC angle of 90°, we continued the unconstrained optimization on the S_0_ PES to obtain the complete isomerization pathway. The optimization shows a gradual decrease of the CNNC angle to 0°, and of the NNC angle to 122° (Figures 5 and S8 A). In contrast, the NNC angle first increases from ≈120° to ≈130° to finally equilibrate at 127° (Figure 5). The changes in the CNN and NNC angles in the ground‐state optimization indicate that the molecule initially attempts to adopt a twisted conformation, which apparently is not accessible, and thus it moves towards the orthogonal conformation, which is stabilized by the lone‐pair⋅⋅⋅π interaction.

Overall, the *E*→*Z* photoisomerization from S_1_ is dominated by the CNNC‐torsional reaction coordinate. However, this torsional motion appears to be assisted by significant changes along the inversion‐reaction coordinate (Δ>15° for ∡CNN and ∡NNC). These changes essentially adjust the relative position of the phenyl and the thiophenyl rings, and result in the adoption of the orthogonal configuration by the (*Z*)‐isomer. Furthermore, this additional degree of freedom is most likely the reason for the observed (Figure 3 B) slightly longer S_1_ lifetime of (*E*)‐TPhAB compared with (*E*)‐AB6e (lifetime distributions centered at 950 fs and 700 fs, respectively).

### Isomerization Mechanism of (Z)‐TphAB‐1

The unconstrained relaxation of (*Z*)‐TphAB‐1 from the Franck–Condon region in S_1_ is initially dominated by a large opening motion of the NNC angle, which increases by ≈35° to ≈158° (Figures 6 and S9). During the last 5° of this opening, the torsion of the CNNC moiety is activated and the CNNC angle quickly reaches 30°. The initial changes of the CNN angles are minor (<5°). The following geometric changes towards the conical intersection with the ground state are governed by the torsion of the CNNC moiety, which brings the CNNC angle from 30° to 90°. This torsional motion is accompanied by a decrease in the NNC angle to 130°. From the conical intersection, the unconstrained optimization proceeds on the ground state towards the (*E*)‐isomer. This relaxation is associated with a decrease of both the CNN and the NNC angles to 115° and a concomitant increase of the CNNC angle to 180°. Similar to the *E*→Z photoisomerization direction, the *Z*→*E* direction is also dominated by the torsional motion about the CNNC moiety. However, here the inversion‐reaction coordinate plays an even more important role, as we detected much larger changes in the CNN and NNC angles (Δ=25° and Δ=42°, respectively) than during the transformation of the (*E*)‐ to the (*Z*)‐isomer.

This photoisomerization mechanism of (*Z*)‐TphAB‐1 can be straightforwardly explained considering the orthogonal geometry of the isomer and the lone‐pair⋅⋅⋅π interaction (see above). Essentially, to initiate the isomerization, first a disruption of the lone‐pair⋅⋅⋅π interaction is required. Such a disruption would then effectively free the torsional reaction coordinate. The disruption of the lone‐pair⋅⋅⋅π interaction is achieved via the opening of the NNC angle, which pulls the S‐atom of the thiophenyl ring away from the plane of the phenyl ring. Therefore, the initial motion on the S_1_ PES is led by the NNC opening. Apparently, above an NNC angle of 150°, the strength of the lone‐pair⋅⋅⋅π interaction is sufficiently reduced and the CNNC torsion is activated. From this point, the mechanism is governed by the torsional reaction coordinate, while the CNN and the NNC moieties work towards planarization of the TphAB molecule. The complex changes that (*Z*)‐TphAB‐1 undergoes along the S_1_ PES also explain the strongly nonexponential dynamics observed in the transient absorption data (sub‐250 fs timescale) discussed above. Such nonexponential dynamics is not present in the relaxation of (*Z*)‐AB.6e

## Conclusion

The uncommon properties of azoheteroarenes have drawn significant attention as an alternative design to the popular AB.^22^ However, those studies have focused mostly on the investigation of nitrogen‐based azoheteroarenes. Here we present a detailed study on the photochromism of a different TphAB photoswitch. We show that the TphAB photoswitch has outstandingly high photoisomerization QYs (QY_*E*(π→π*)→*Z*_=44 %, QY_*Z*(n→π*)→*E*_=65 %.), photoconversion levels (PSS_365_ contains only ≈3 % (*E*)‐isomer, while PSS_285_ contains only ≈13 % (*Z*)‐isomer), and fatigue resistance. Our theoretical calculations demonstrate that the (*Z*)‐isomer of TphAB adopts a geometry where the thiophene ring is perfectly orthogonal to the phenyl ring, with the S‐atom facing the phenyl ring. This orthogonal geometry is stabilized by a rare lone‐pair⋅⋅⋅π interaction between the S‐atom and the phenyl ring. We reveal that while the ultrafast photoisomerization of TphAB occurs on a timescale similar to that of AB, the corresponding dynamics is remarkably rich. The torsional motion about the CNNC moiety is the dominant reaction coordinate. However, the formation and disruption of the unusual orthogonal geometry of the (*Z*)‐isomer requires significantly larger changes along the inversion coordinate (∡CNN and ∡NNC) than those discussed in AB.10h Therefore, the presence of the lone‐pair⋅⋅⋅π interaction and the ensuing orthogonal geometry add an additional degree of freedom compared to AB, which evidently alters the photochemistry of TPhAB.

Our work delivers important insight into the molecular basis of the photoisomerization mechanism operating in TphAB, which is relevant not only to azoheteroarenes but also to conventional ABs. We also establish TphAB as an excellent photoswitch with versatile properties, in many ways better than the once widely utilized conventional AB and many of its derivatives.

## Conflict of interest

The authors declare no conflict of interest.

## Supporting information

As a service to our authors and readers, this journal provides supporting information supplied by the authors. Such materials are peer reviewed and may be re‐organized for online delivery, but are not copy‐edited or typeset. Technical support issues arising from supporting information (other than missing files) should be addressed to the authors.

Supplementary
