# Supplementary material for: Thiophenylazobenzene: An Alternative Photoisomerization Controlled by Lone‐Pair⋅⋅⋅π Interaction
Source: Angew Chem Int Ed Engl. 2019 Nov 18;59(1):380–7. doi: 10.1002/anie.201909739 (PMC6973119; doi:10.1002/anie.201909739)
Supplement: Supplementary file 1 — Supplementary [file ANIE-59-380-s001.pdf]

Angewandte Chemie
Eine Zeitschrift der Gesellschaft Deutscher Chemiker

Supporting Information

Thiophenylazobenzene: an Alternative Photoisomerization Controlled by Lone-Pair···π Interaction

Chavdar Slavov[+], Chong Yang[+], Andreas H. Heindl, Hermann A. Wegner, Andreas Dreuw, and Josef Wachtveitl**

anie_201909739_sm_miscellaneous_information.pdf

Table of Contents

Synthesis of Thiophenylazobenzene 1

Stationary UV/vis experiments 3

UV/vis pump-probe 3

Data analysis 3

Computational details 3

References 4

Synthesis of Thiophenylazobenzene

Chemicals were used as purchased from Sigma-Aldrich, Acros Organics, Alfa Aesar and TCI Europe. Anhydrous solvents were purchased from Acros Organics. Technical grade solvents used during workup and purification were distilled prior to use. Air and/or water-sensitive reactions were carried out under Schlenk-conditions. Solids were dried under high vacuum when necessary. Flash column chromatography and column chromatography was carried out with Silica 60 M (0.04 – 0.063 mm) from Macherey Nagel GmbH & Co. KG. Thin layer chromatography was carried out on Polygram® SIL G/UV254 from Macherey Nagel GmbH & Co. KG. NMR spectra were measured on a Bruker Avance II 200 MHz, Avance II 400 MHz or Avance III 600 MHz spectrometer at room temperature. Chemical shifts are reported in parts per million (ppm) relative to the solvent peak, coupling constants (J) are reported in Hertz (Hz). Deuterated solvents were obtained from Deutero GmbH (Kastellaun, Germany) or Euriso − Top GmbH. For all azobenzenes, the thermodynamically more stable E-isomer is reported. ESI-MS spectra were performed on a Bruker Daltonics Micro TOF. Melting points were measured on a Krüss M5000 melting point meter.

Synthesis of 1-(thiophen-2-yl)-2-(*p*-tolyl)diazene **2**.

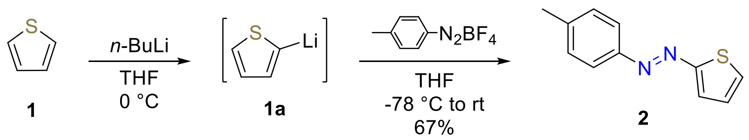

Synthesis of 4-methylphenyldiazonium tetrafluoroborate. *p*-Toluidine (2.18 g, 20.1 mmol, 1.00 eq.) was suspended in water (10 mL) and aq. HBF_4 (50%, 8. mL, 64 mmol, 3.2 eq.) was added. The suspension was cooled to 0 °C and a solution of $NaNO_2$ (1.41 g, 20.4 mmol, 1.01 eq.) in water (5 mL) was added dropwise. The suspension was vigorously stirred at 0 °C for 45 min. Following, the precipitate was filtered off, washed with plenty of Et_2O and was dried in vacuum to yield a pearly grey solid (3.22 g, 78%). The product was used without further purification.

Azo coupling. To a solution of thiophene (1.2 mL, 15 mmol, 1.0 eq.) in dry THF (15 mL) under a nitrogen atmosphere, *n*-BuLi in hexanes (10 mL, ca. 1.6 M, 16 mmol, 1.1 eq.) was added dropwise at 0 °C. The orange solution was stirred at this themperature for 30 min and was then transferred dropwise to a suspension of 4-methylphenyldiazonium tetrafluoroborate (2.99 g, 14.5 mmol, 1.00 eq.) in dry THF (15 mL) at –78 °C under N_2. Following, the dark red solution was stirred while being allowed to warm to rt for 1 h. Afterwards, the reaction was quenched by adding sat. aq. $NaHCO_3$ (30 mL) and EtOAc (50 mL). After phase separation, the organic layer was washed with brine (50 mL), was dried over $MgSO_4$, filtered and the solvents were evaporated under reduced pressure. The residue was filtered over a silica plug (DCM wash), concentrated, and was purified by flash column chromatography (SiO_2, cyclohexane/EtOAc; 100:1, 50:1) to yield a brown to red solid, which was recrystallized from EtOH/H_2O (2:1) to yield a bronze or yellow crystalline solid (1.98 g, 67%).

^{1}H NMR (400 MHz, $CDCl_3$) δ / ppm: 7.81 – 7.73 (m, 3H), 7.38 (dd, J = 5.4, 1.3 Hz, 1H), 7.30 – 7.27 (m, 2H), 7.18 – 7.12 (m, 1H), 2.42 (s, 3H).
^{13}C NMR (101 MHz, $CDCl_3$) δ / ppm: 160.7, 150.3, 141.5, 131.2, 129.9, 128.1, 127.5, 123.0, 21.7.
HRMS (ESI): m/z for $C_{11}H_{10}N_{2S}Na^+$; calcd. 225.0457, found 225.0461.
TLC (SiO_2, cyclohexane/EtOAc; 9:1): R_f = 0.45.
m.p. 113 °C.

^{1}H and $^{13}C\{^{1}H\}$ NMR spectra

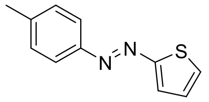

2

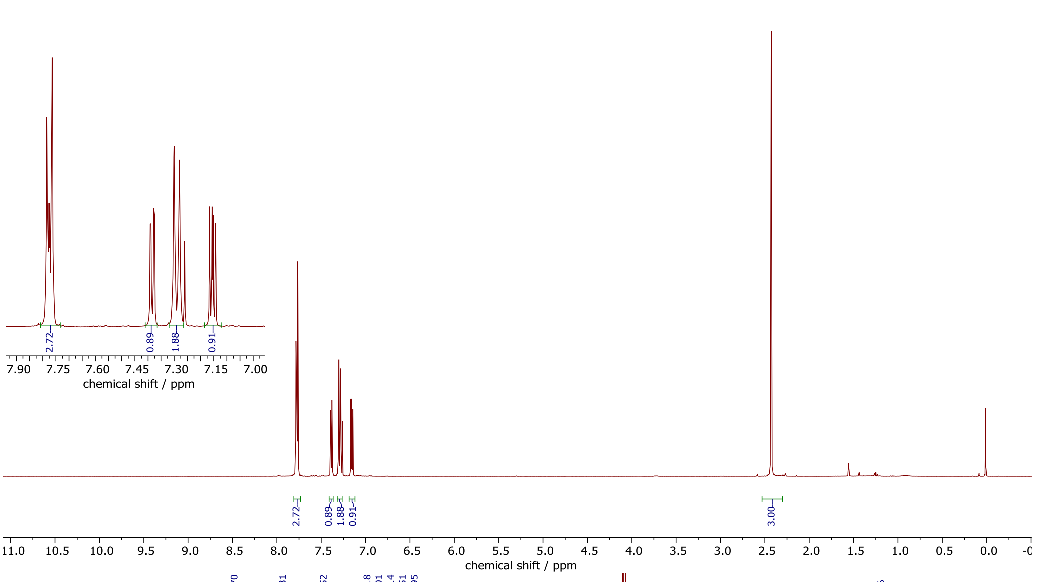

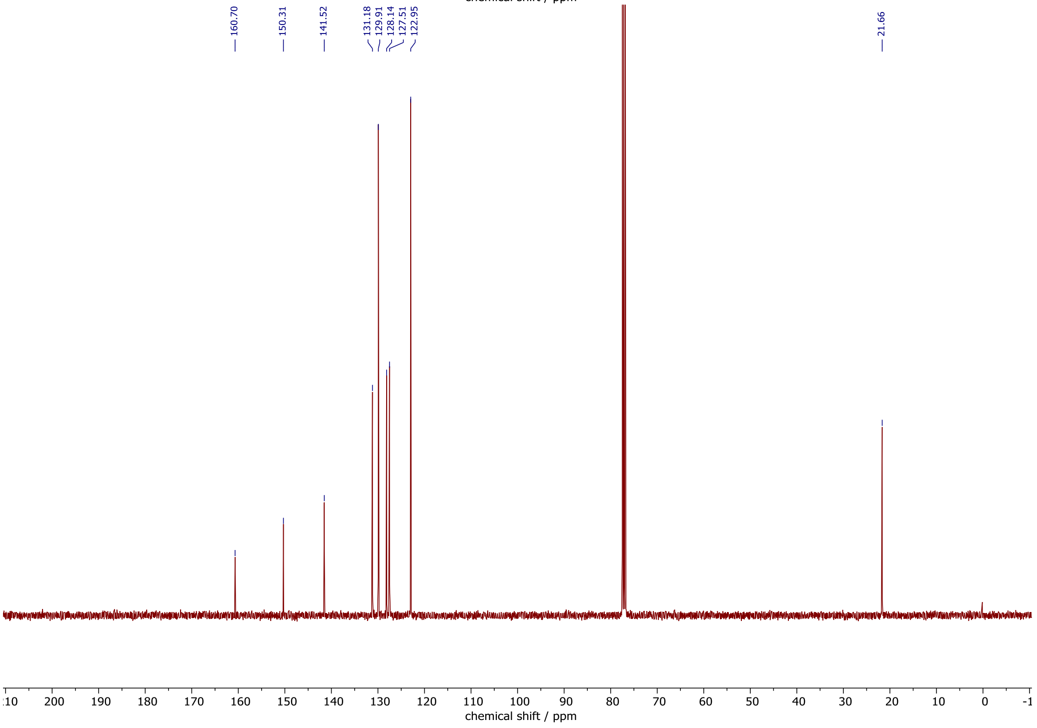

Stationary UV/vis experiments

The steady state absorption spectra and the irradiation experiments were performed in acetonitrile and using a standard spectrophotomer (Specord S600, Analytik Jena). The PSS were generated as follows: i) PSS_{365} was achieved by using a 365 nm LED (ThorLabs M365L2); ii) the PSS_{285} was achieved by using a 285 nm LED (ThorLabs M285L5). The QY of the $E \rightarrow Z$ isomerization after ππ* excitation of the compounds was determined approximately using the above mentioned spectrophotometer and LEDs and as described in ref.[1]

UV/vis pump-probe

The setup for time-resolved transient absorption measurements was described previously in detail.[1] It consists of an oscillator/amplifier system (Clark, MXR-CPA-iSeries, 1 kHz, 775 nm 150 fs) which provides the ultrashort laser fundamental pulses. These pulses are then used to drive a home-built two stage NOPA (noncollinear optical parametric amplifier)[2] system and a sum frequency generation stage to produce the excitation pulses used in the experiments. These NOPA pulses were pre-compressed using a prism compressor placed between the two NOPA stages. In addition, part of the laser fundamental was focused into a CaF_2-crystal (5 mm) to generate light continuum for the probe pulses. The cross-correlation between the excitation pulses and the probe pulses was ~100 fs. The signal was detected in reference mode by two spectrometers (600 lines/mm gratings, blazed at 300 nm, and a 64-channel photodiode array). Anisotropic contributions were eliminated by measuring under magic angle conditions (54.7° pump-probe polarization difference). The experiments were carried out in a fused silica cuvette with 1 mm optical path length, which was continuously moved to ensure fresh sample volume for each laser excitation pulse. In addition, the sample was continuously irradiated with a 285 nm LED for the (*E*)-isomer experiments and with a 365 nm LED for the (*Z*)-isomer experiments to prevent accumulation of the opposite isomer during the experiments. The 365 nm LED irradiation results in dominant population (~90%) of the (*Z*)-isomer, so that the $Z \rightarrow E$ experiments can be conducted at 445 nm excitation.

Data analysis

The analysis of the experimental data was performed using OPTIMUS (www.optimusfit.org).[3] Lifetime distribution analysis (LDA) was used for the ultrafast transient absorption data as a model independent method of analysis that naturally deals with non-exponential or distributed kinetics. In this analysis, the pre-exponential amplitudes of a set of 100 exponential functions with fixed, equally spaced (on a decimal logarithm scale) lifetimes are determined. The obtained pre-exponential amplitudes at each detection wavelength can be presented in the form of a contour lifetime density map (LDM)[4]. The transient absorption data around time zero position contain the so-called coherent artifact contributions. These were approximated with a function composed of a Gaussian and/or its first and second derivative[3, 5] and fitted within the same routine as the exponential fitting.

Computational details

The ground state geometries for two possible TphAB in both (*E*)- and (*Z*)-configurations were optimized by employing density functional theory (DFT/BHLYP)[6]. Time-dependent DFT[7] has proved to be able to reproduce accurate excited state calculations for AB derivatives[8]. Using time-dependent DFT in combination with conductor-like polarizable continuum model (C-PCM)[9] for acetonitrile solvation ($\varepsilon = 37.5$), we performed electronic structure and excitation energy calculations of TphAB and computed their UV/vis absorption spectra at the BHLYP/6-31+G*[10] level of theory. We used the algebraic diagrammatic construction scheme (ADC(2))[11] to benchmark the results of TDDFT calculations. To simulate the $E \rightarrow Z$, $Z \rightarrow E$ isomerization process, Tamm-Dancoff approximation (TDA)[12] and the state-tracking technique[13] was applied to the unconstrained geometry optimization of (*E*)- and (*Z*)-TphAB-1. In most cases, the 6-31+G* basis set was adopted, while a smaller basis set 6-31G* was used in the unconstrained geometry optimization. In the case of $E \rightarrow Z$ direction, the uncontained optimization converges to a local minimum at CNNC of 116° due to the present of a barrier on the potential energy surface. From this point, constrained optimizations were carried out for CNNC between 116° and 90° to reach the conical intersection with the grounds state. From CNNC 90°, again unconstrained optimization was carried out in the ground state. All calculations were performed with the Q-Chem 5.0[14] package.

References

[1] C. Slavov, et al., Phys. Chem. Chem. Phys. **2015**, 17, 14045-14053.
[2] a) T. Wilhelm, J. Piel, E. Riedle, Opt. Lett. **1997**, 22, 1494-1496; b) E. Riedle, M. Beutter, S. Lochbrunner, J. Piel, S. Schenkl, S. Spörlein, W. Zinth, Appl. Phys. B **2000**, 71, 457-465.
[3] C. Slavov, H. Hartmann, J. Wachtveitl, Anal. Chem. **2015**, 87, 2328-2336.
[4] R. Croce, M. G. Müller, R. Bassi, A. R. Holzwarth, Biophys. J. **2001**, 80, 901-915.
[5] S. A. Kovalenko, A. L. Dobryakov, J. Ruthmann, N. P. Ernsting, Phys. Rev. A **1999**, 59, 2369-2384.
[6] A. D. Becke, J. Chem. Phys. **1993**, 98, 1372-1377.
[7] a) E. Runge, E. K. U. Gross, Phys. Rev. Lett. 1984, 52, 997-1000; b) M. E. Casida, Recent advances in density functional methods, Vol. Part I, World Scientific, **1995**; c) A. Dreuw, M. Head-Gordon, Chem. Rev. **2005**, 105, 4009-4037.
[8] a) C. R. Crecca, A. E. Roitberg, J. Phys. Chem. A **2006**, 110, 8188-8203; b) C. Yang, C. Slavov, H. A. Wegner, J. Wachtveitl, A. Dreuw, Chem. Sci. **2018**, 9, 8665-8672; c) C. Slavov, C. Yang, A. H. Heindl, T. Stauch, H. A. Wegner, A. Dreuw, J. Wachtveitl, J. Phys. Chem. Lett. **2018**, 9, 4776-4781.
[9] a) J. Liu, W. Liang, J. Chem. Phys. **2011**, 135, 014113; b) J. Liu, W. Liang, J. Chem. Phys. **2011**, 135, 184111; c) J. Liu, W. Liang, J. Chem. Phys. **2013**, 138, 024101.
[10] W. J. Hehre, R. Ditchfield, J. A. Pople, J. Chem. Phys. **1972**, 56, 2257-2261.
[11] a) J. Schirmer, Phys. **Rev**. A **1982**, 26, 2395-2416; b) M. Wormit, D. R. Rehn, P. H. P. Harbach, J. Wenzel, C. M. Krauter, E. Epifanovsky, A. Dreuw, Mol. Phys. 2014, 112, 774-784; c) A. Dreuw, M. Wormit, WIREs Comput. Mol. Sci. **2015**, 5, 82-95.
[12] S. Hirata, M. Nooijen, R. J. Bartlett, Chem. Phys. Lett. **2000**, 326, 255-262.
[13] K. D. Closser, O. Gessner, M. Head-Gordon, J. Chem. Phys. **2014**, 140, 134306.
[14] Y. Shao, et al., Mol. Phys. **2015**, 113, 184-215.

Table S1. Excitation energy and oscillator strength for (*E*)- and (*Z*)-isomers of the two possible TphAB conformations (TphAB-1 and TphAB-1).

	BHHLYP / 6-31+g* / C-PCM				ADC(2) / 6-31+g*			
	(E)-TphAB-1		***(E)-TphAB-2***		***(E)-TphAB-1***		***(E)-TphAB-2***	
Excited state	Excitation energy (eV)	strength	Excitation energy (eV)	strength	Excitation energy (eV)	strength	Excitation energy (eV)	strength
1	3.1103	0.0000	3.0280	0.0002	3.0239	0.0000	2.9718	0.0001
2	3.5620	0.9463	3.5448	0.9750	3.9074	0.7787	3.8984	0.8653
3	4.6788	0.0232	4.6621	0.0297	4.6022	0.0685	4.6011	0.0064
4	4.7382	0.1263	4.8529	0.0705	4.6576	0.1259	4.7214	0.0616
5	5.1443	0.0103	5.0694	0.0094	5.1701	0.0085	5.0972	0.0148
6	5.7430	0.1190	5.7104	0.1882	5.8846	0.1455	5.8659	0.1916
7	6.0048	0.0992	6.0040	0.0231	5.9096	0.0010	5.9147	0.0029
8	6.0162	0.0008	6.0347	0.0056	5.9311	0.0001	5.9702	0.0029
9	6.2337	0.0054	6.0898	0.0001	6.0489	0.0145	6.0884	0.0000
10	6.5441	0.0065	6.4103	0.0656	6.0895	0.0002	6.1247	0.0000
Total energy (a.u.)	**-932.5127**		**-932.5077**		**-930.6046**		**-930.5998**	
	(Z)-TphAB-1		***(Z)-TphAB-2***		***(Z)-TphAB-1***		***(Z)-TphAB-2***	
Excited state	Excitation energy (eV)	strength	Excitation energy (eV)	strength	Excitation energy (eV)	strength	Excitation energy (eV)	strength
1	3.0875	0.0025	2.9038	0.0493	3.0583	0.0022	2.9342	0.0298
2	4.4378	0.3374	4.2798	0.2770	4.5885	0.1162	4.5527	0.3082
3	4.4940	0.0052	4.5119	0.1191	4.6735	0.0024	4.6246	0.0136
4	4.9510	0.2459	4.8980	0.1102	4.9415	0.0116	4.8276	0.0258
5	5.3633	0.0362	5.1131	0.0497	5.0537	0.3897	4.9731	0.0498
6	5.3834	0.0157	5.4812	0.0686	5.2814	0.0804	5.3793	0.0753
7	5.6854	0.1408	5.7268	0.0605	5.5022	0.0001	5.4821	0.0521
8	6.2819	0.0508	6.0269	0.0030	5.5521	0.0231	5.5728	0.0145
9	6.3778	0.0050	6.2029	0.0112	5.7862	0.0042	5.6602	0.0130
10	6.4241	0.0821	6.2205	0.0059	6.0170	0.0164	5.9277	0.0322
total energy (a.u.)	**-932.4882**		**-932.4848**		**-930.5858**		**-930.5816**	

Table S2. Unconstrained/partially constrained optimization of (*E*)-TphAB-1 from the S_1 Franck-Condon region. The optimization was performed at the BHHLYP / 6-31G* / C-PCM level of theory. Unconstrained optimization (**dark green**) and constrained optimization **(light green**) on the S1 state; unconstrained optimization on the ground state (**blue**).

CNN	NNC	CNNC	GS Energy	Relative E (a.u.)	Relative E (eV)	1st	2nd	3rd	4th	GS+1st	GS+2nd	GS+3rd	GS+4th
116.21	115.717	180	-932.4943282	0	0	3.1368	3.6205	4.8091	4.8883	3.1368	3.6205	4.8091	4.8883
127.447	127.608	179.8	-932.4762758	0.018052	0.491222	2.2377	3.5985	4.7723	4.8408	2.728922	4.089722	5.263522	5.332022
128.872	129.272	179.362	-932.4733984	0.02093	0.569519	2.1047	3.5245	4.7497	4.8116	2.674219	4.094019	5.319219	5.381119
129.419	130.037	178.057	-932.4718104	0.022518	0.612731	2.0548	3.5086	4.7375	4.7931	2.667531	4.121331	5.350231	5.405831
129.481	130.342	175.082	-932.4711851	0.023143	0.629745	2.034	3.513	4.7373	4.7919	2.663745	4.142745	5.367045	5.421645
128.765	129.521	159.167	-932.4683933	0.025935	0.705714	1.9003	3.5872	4.6606	4.7084	2.606014	4.292914	5.366314	5.414114
127.686	128.549	143.076	-932.4607797	0.033548	0.912886	1.6052	3.6683	4.526	4.5713	2.518086	4.581186	5.438886	5.484186
126.059	126.761	126.801	-932.4479821	0.046346	1.261122	1.1998	3.7116	4.2671	4.347	2.460922	4.972722	5.528222	5.608122
125.128	125.538	117.38	-932.4385085	0.05582	1.518907	0.9332	3.6519	4.084	4.1957	2.452107	5.170807	5.602907	5.714607
125.56	125.38	116.889	-932.4379268	0.056401	1.534738	0.9163	3.6322	4.0864	4.1893	2.451038	5.166938	5.621138	5.724038
129.919	125.25	116.381	-932.4373567	0.056971	1.550251	0.9006	3.6185	4.0881	4.1863	2.450851	5.168751	5.638351	5.736551
125.875	125.165	116.115	-932.4370358	0.057292	1.558981	0.8918	3.6194	4.0804	4.1812	2.450781	5.178381	5.639381	5.740181
125.884	125.173	116.125	-932.4370308	0.057297	1.559119	0.8916	3.6215	4.0806	4.1819	2.450719	5.180619	5.639719	5.741019
126.479	124.667	110	-932.4290295	0.065299	1.776842	0.6755	3.5774	3.9621	4.082	2.452342	5.354242	5.738942	5.858842
127.762	123.877	105	-932.4220256	0.072303	1.967424	0.4863	3.5399	3.8581	3.9907	2.453724	5.507324	5.825524	5.958124
128.328	123.627	104	-932.4205129	0.073815	2.008586	0.4446	3.5361	3.8363	3.9708	2.453186	5.544686	5.844886	5.979386
130.068	123.123	102	-932.4172159	0.077112	2.098303	0.3502	3.5445	3.7878	3.9272	2.448503	5.642803	5.886103	6.025503
131.183	122.959	101	-932.4154585	0.07887	2.146123	0.2971	3.5606	3.7571	3.9007	2.443223	5.706723	5.903223	6.046823
132.14	122.967	100.5	-932.4136895	0.080639	2.194259	0.2403	3.5808	3.7181	3.8689	2.434559	5.775059	5.912359	6.063159
132.102	122.959	100	-932.4137319	0.080596	2.193104	0.2417	3.5794	3.7192	3.8697	2.434804	5.772504	5.912304	6.062804
131.7	118.966	90	-932.4095311	0.084797	2.307414	0.0926	3.579	3.7115	3.7594	2.400014	5.886414	6.018914	6.066814
136.499	119.794	77.892	-932.4190305	0.075298	2.048923	0.6567	3.7175	3.9766	4.0648	2.705623	5.766423	6.025523	6.113723
127.71	122.643	64.6	-932.4329161	0.061412	1.671084	1.1891	3.7178	4.1689	4.3711	2.860184	5.388884	5.839984	6.042184
126.468	126.669	49.776	-932.4437234	0.050605	1.377005	1.7822	3.8437	4.3657	4.6426	3.159205	5.220705	5.742705	6.019605
131.042	131.935	39.966	-932.4519412	0.042387	1.153391	2.0503	4.0958	4.6242	4.7861	3.203691	5.249191	5.777591	5.939491
127.316	131.707	32.475	-932.4579213	0.036407	0.990668	2.3646	4.1773	4.6997	4.9166	3.355268	5.167968	5.690368	5.907268
129.049	128.902	22.749	-932.4627425	0.031586	0.859476	2.6254	4.1908	4.7491	4.9935	3.484876	5.050276	5.608576	5.852976
126.057	128.385	16.861	-932.4658853	2.84E-02	0.77396	2.8194	4.237	4.7749	5.0244	3.59336	5.01096	5.54886	5.79836
124.621	128.229	11.812	-932.4678884	2.64E-02	0.719451	2.9588	4.3024	4.8329	5.0149	3.678251	5.021851	5.552351	5.734351
123.398	127.661	7.927	-932.4690959	0.025232	0.686594	3.0578	4.369	4.8479	5.0293	3.744394	5.055594	5.534494	5.715894

122.423	127.081	5.356	-932.4697131	0.024615	0.669799	3.1442	4.4506	4.8033	5.0976	3.813999	5.120399	5.473099	5.767399
122.771	127.635	3.169	-932.4699564	0.024372	0.66318	3.1475	4.5053	4.7948	5.1584	3.81068	5.16848	5.45798	5.82158
122.449	127.142	2.045	-932.4701287	0.024199	0.658491	3.1818	4.5622	4.764	5.2009	3.840291	5.220691	5.422491	5.859391
122.358	127.188	0.025	-932.4701978	0.02413	0.656612	3.1899	4.5868	4.7488	5.212	3.846512	5.243412	5.405412	5.868612
122.529	127.302	0.15	-932.4702187	0.024109	0.656043	3.1811	4.5796	4.7519	5.2125	3.837143	5.235643	5.407943	5.868543
122.438	127.268	-0.119	-932.4702263	0.024102	0.655836	3.1833	4.5762	4.7486	5.2088	3.839136	5.232036	5.404436	5.864636
122.468	127.252	-0.461	-932.4702288	0.024099	0.655769	3.1824	4.5764	4.7484	5.2082	3.838169	5.232169	5.404169	5.863969
122.476	127.261	-0.53	-932.4702295	0.024099	0.655748	3.1819	4.5763	4.7485	5.2083	3.837648	5.232048	5.404248	5.864048
122.487	127.278	-0.478	-932.4702302	0.024098	0.655731	3.1812	4.5759	4.7483	5.2084	3.836931	5.231631	5.404031	5.864131

Table S3. Unconstrained optimization of (Z)-TphAB-1 from the S1 Franck-Condon region. The optimization was performed at the BHHLYP / 6-31G* / C-PCM level of theory. Optimization on the S_1 state (green); optimization on the ground state (blue).

CNN	NNC	CNNC	GS Energy	Relative E (a.u.)	Relative E (eV)	1st	2nd	3rd	4th	GS+1st	GS+2nd	GS+3rd	GS+4th
127.402	122.587	0.073	-932.4701415	0.024376395	0.663306087	3.1465	4.5713	4.5953	5.0988	3.809806	5.234606	5.258606	5.762106
135.479	135.643	0.101	-932.454018	0.040499892	1.102042561	2.322	4.4886	4.4896	5.0873	3.424043	5.590643	5.591643	6.189343
134.29	141.477	0.149	-932.4450811	0.049436812	1.345225083	1.9478	4.3801	4.4818	4.9889	3.293025	5.725325	5.827025	6.334125
132.885	143.762	0.006	-932.4428257	0.05169219	1.40659619	1.8692	4.3435	4.4896	4.9555	3.275796	5.750096	5.896196	6.362096
129.817	147.522	0.229	-932.439893	0.054624837	1.486396431	1.7734	4.2839	4.518	4.9197	3.259796	5.770296	6.004396	6.406096
126.876	150.805	1.224	-932.4373555	0.057162419	1.555446581	1.6951	4.2422	4.5537	4.8853	3.250547	5.797647	6.109147	6.440747
125.524	152.597	3.202	-932.4358409	0.058677008	1.596660067	1.6491	4.2275	4.5743	4.8727	3.24576	5.82416	6.17096	6.46936
124.955	153.775	6.736	-932.4345272	0.05999069	1.632406663	1.6098	4.2228	4.5851	4.8643	3.242207	5.855207	6.217507	6.496707
124.06	155.912	16.794	-932.4311211	0.063396802	1.725090387	1.5064	4.2057	4.5915	4.8255	3.23149	5.93079	6.31659	6.55059
123.486	157.636	27.342	-932.4270859	0.06743196	1.834891074	1.3873	4.1812	4.581	4.7804	3.222191	6.016091	6.415891	6.615291
125.162	156.816	39.699	-932.4231843	0.071333536	1.941056859	1.268	4.1589	4.536	4.7631	3.209057	6.099957	6.477057	6.704157
126.028	155.693	49.961	-932.4194708	0.07504704	2.042105016	1.142	4.1341	4.4809	4.7083	3.184105	6.176205	6.523005	6.750405
121.355	143.692	51.813	-932.4304491	0.06406873	1.743374217	1.2638	4.0229	4.303	4.5273	3.007174	5.766274	6.046374	6.270674
122.833	138.697	62.652	-932.4264005	0.068117351	1.853541227	1.0081	3.9047	4.1078	4.3614	2.861641	5.758241	5.961341	6.214941
110.336	140.844	65.33	-932.4061192	0.088398689	2.405416735	0.7727	3.4794	3.9284	4.0003	3.178117	5.884817	6.333817	6.405717
115.957	135.54	76.826	-932.4116189	0.082898938	2.255763013	0.5015	3.3677	3.7505	3.8188	2.757263	5.623463	6.006263	6.074563
117.179	130.42	90.817	-932.4045899	0.08992798	2.447030269	0.0404	3.0904	3.5973	3.689	2.48743	5.53743	6.04433	6.13603
117.179	130.42	90.817	-932.4045899	0.089927936	2.447029058	0.0681	3.2453	3.602	3.76	2.515129	5.692329	6.049029	6.207029
116.878	134.074	104.78	-932.4194365	0.075081415	2.043040384	0.6693	3.4729	3.9943	4.1247	2.71234	5.51594	6.03734	6.16774
115.78	124.089	116.25	-932.4351854	0.059332451	1.614495319	1.2234	3.473	4.1276	4.1662	2.837895	5.087495	5.742095	5.780695
114.538	119.54	132.03	-932.4535749	0.040943003	1.114100052	1.936	3.5831	4.3374	4.4136	3.0501	4.6972	5.4515	5.5277
113.265	122.164	147.91	-932.4735658	0.020952123	0.57012823	2.5051	3.7105	4.6766	4.6922	3.075228	4.280628	5.246728	5.262328
114.89	116.942	163.69	-932.4878248	0.006693038	0.182124268	3.0283	3.8056	4.8162	4.8684	3.210424	3.987724	4.998324	5.050524
114.947	116.501	179.94	-932.4939054	0.000612475	0.016666044	3.1844	3.8938	4.8681	4.9819	3.201066	3.910466	4.884766	4.998566
115.092	115.54	179.33	-932.4942625	0.000255366	0.006948767	3.1998	3.8692	4.8489	4.9651	3.206749	3.876149	4.855849	4.972049
115.353	115.625	179.14	-932.4943726	0.000145241	0.003952139	3.1801	3.8504	4.8355	4.9529	3.184052	3.854352	4.839452	4.956852
115.146	115.772	179.86	-932.4945105	7.35E-06	0.000199982	3.1934	3.8515	4.843	4.9604	3.1936	3.8517	4.8432	4.9606
115.252	115.88	179.88	-932.494517	9.25E-07	2.51729E-05	3.1855	3.8506	4.8418	4.9587	3.185525	3.850625	4.841825	4.958725
115.186	115.878	179.96	-932.4945179	0	0	3.1872	3.8489	4.8406	4.9583	3.1872	3.8489	4.8406	4.9583

Table S4. Parameters obtained from the thermal *Z* → *E* relaxation and used in the Eyring calculation.

T [Cº]	T [K]	1/T [1/K]	k [s^{-1}]	$t_{1/2}$ [s]	ln(k/T)
10	283.15	0.00353	2.69E-05	429.5	-16.17
20	293.15	0.00341	9.68E-05	119.3	-14.92
30	303.15	0.00330	3.12E-04	37.0	-13.79
40	313.15	0.00319	9.70E-04	11.9	-12.68

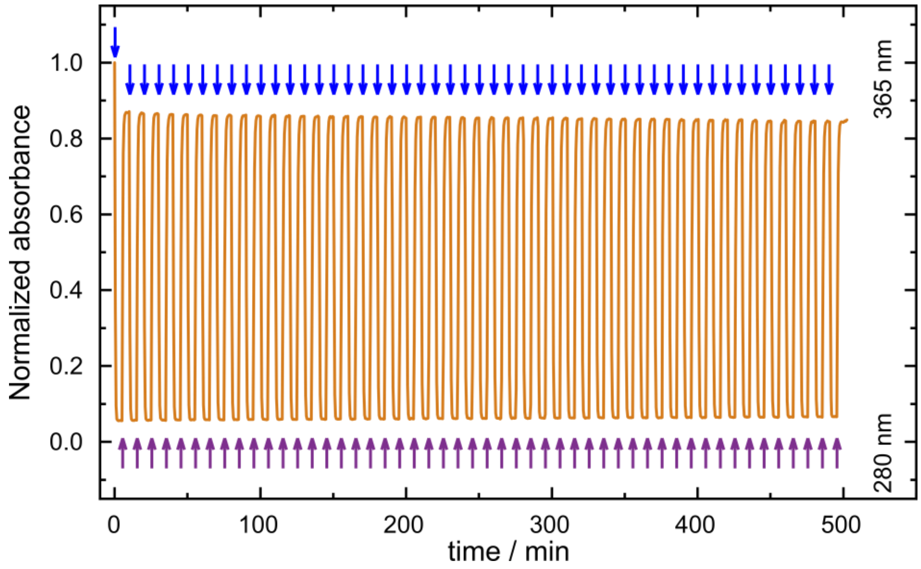

Figure S1. Cyclic conversion of TphAB between its (*E*)- and (*Z*)-isomers achieved by high intensity irradiation at 365 nm and 280 nm in the corresponding ππ* transition bands of the isomers.

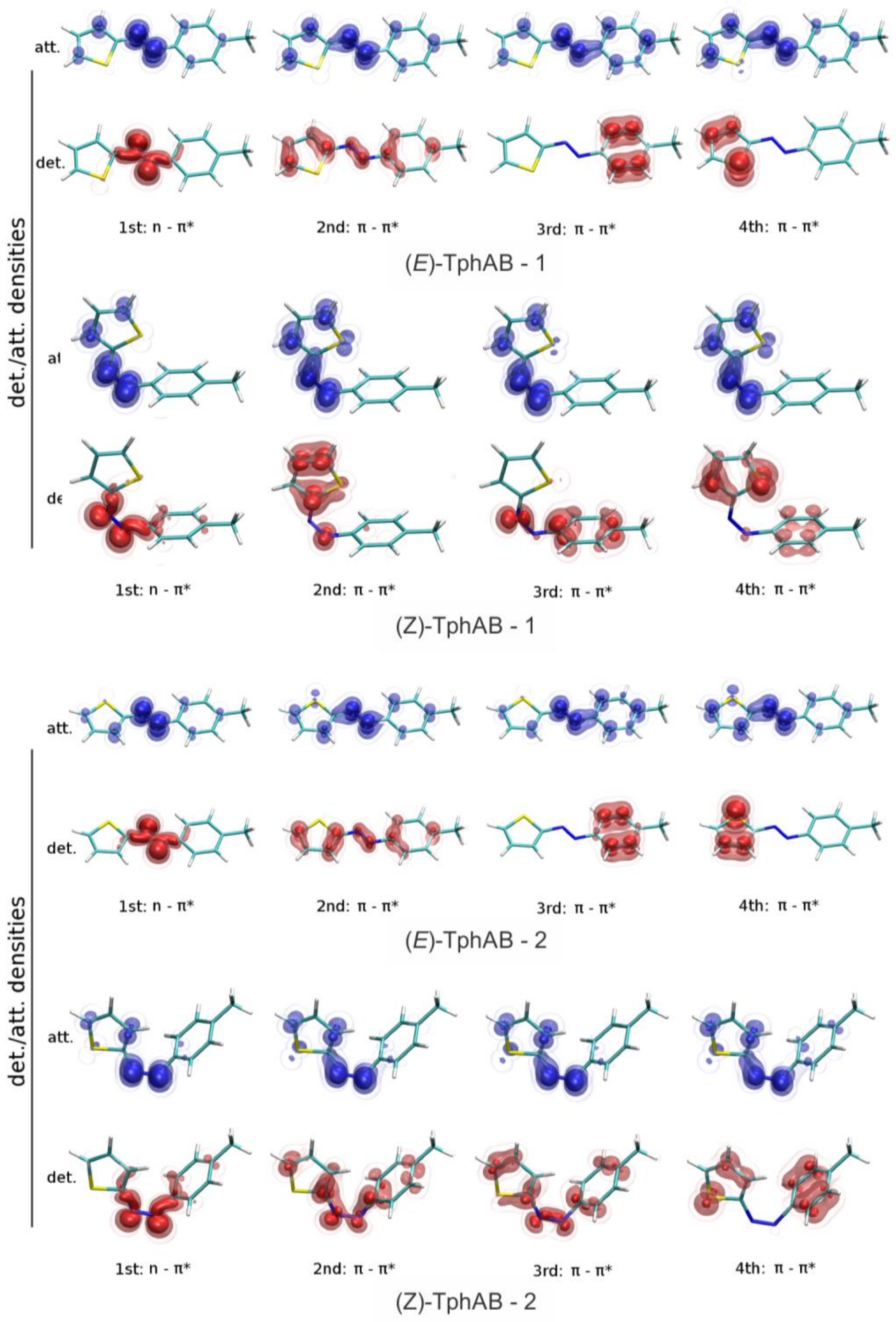

Figure S2. Detachment (**red**) and attachment (**blue**) densities for the lowest excited states at the TDDFT/BHLYP/6-31G* level of theory for the stable ground state geometries of the (*E*)- and (*Z*)-isomer of the studied ThpAB. The detachment density corresponds to that part of the density that is removed upon excitation, while the attachment density is the one that is added.

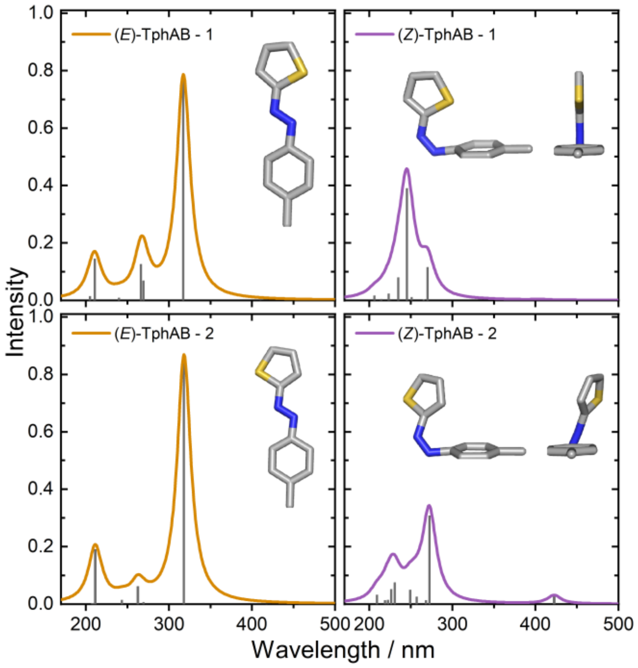

Figure S3. Calculated excitation energies at the ADC level of theory for the stable ground state geometries of the (*E*)- and (*Z*)-isomer of the studied ThpAB.

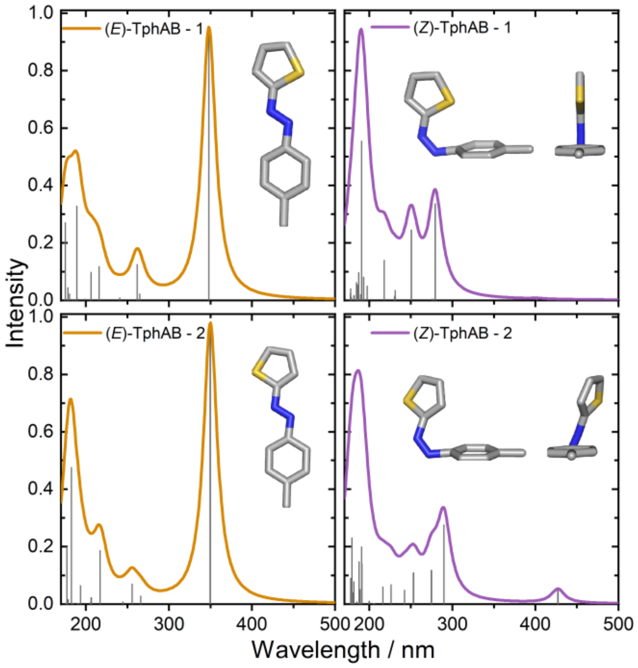

Figure S4. Calculated excitation energies at the BHLYP level of theory for the stable ground state geometries of the (*E*)- and (*Z*)-isomer of the studied ThpAB.

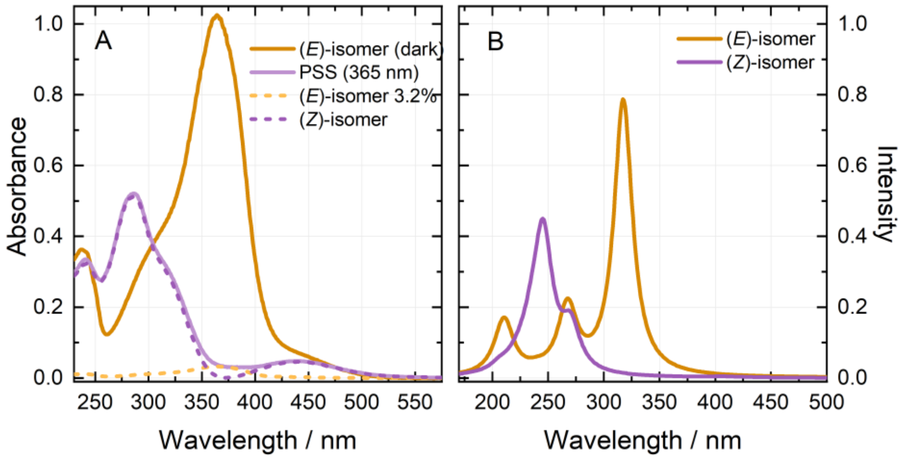

Figure S5. (**A**) Experimental absorption spectra of the (*E*)-isomer, the PSS_{365} and the estimated pure (*Z*)-isomer spectra. Note that the (*Z*)-isomer spectrum is determined based on the highest possible contribution of the (*E*)-isomer to the PSS365 spectrum (i.e. 3.2%). (**B**) Calculated spectra at the ADC level of theory for the (*E*)- and the (*Z*)-isomer of ThpAB. Note that the calculated spectra are a weighted sum of the contribution from the two stable ground state geometries. Therefore, the (*E*)-isomer spectrum is a sum of 99.46% of (*E*)-TphAB-1 and 0.54% of (*E*)-TphAB-2, while the (*Z*)-isomer spectrum is a sum of 97.25% of (*Z*)-TphAB-1 and 2.75% of (*Z*)-TphAB-2.

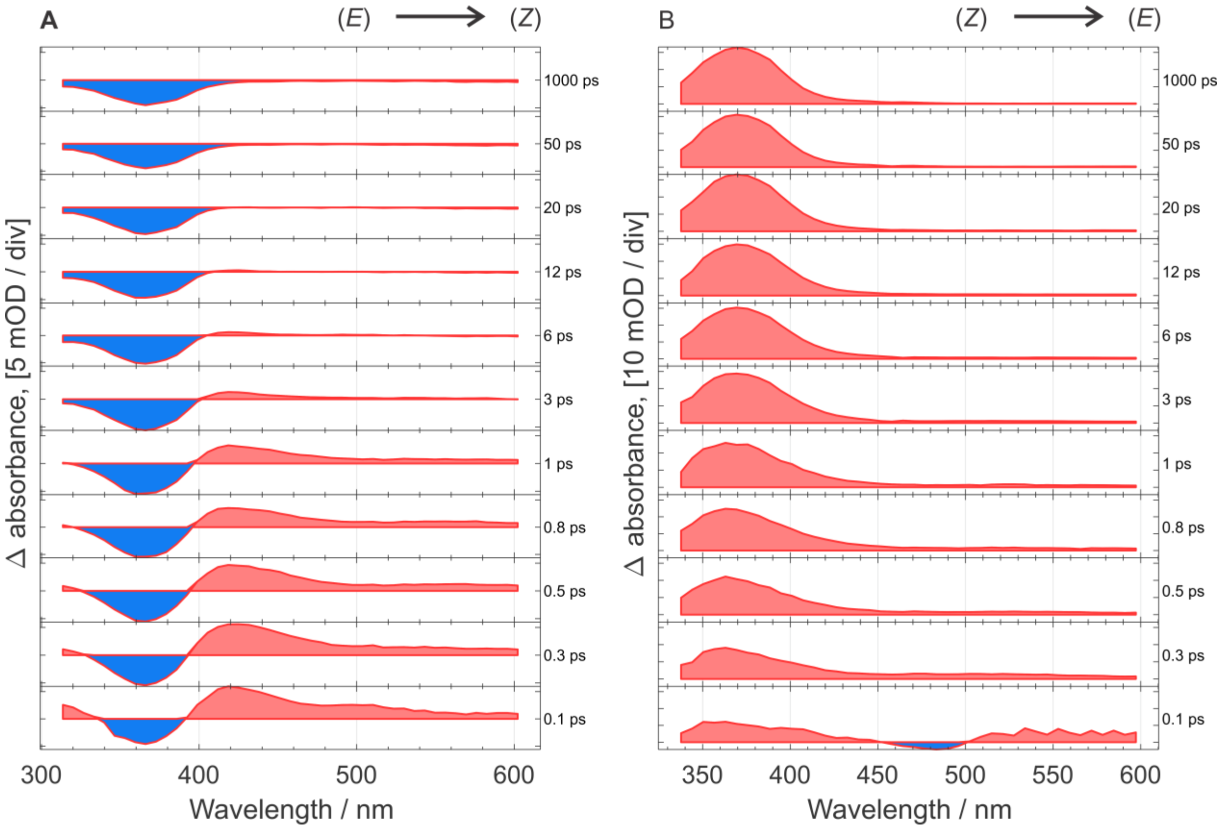

Figure S6. Transient spectra at selected points in time representing the spectral evolution in acetonitrile of (*E*)-TphAB after 355 nm excitation (**A**) and of (*Z*)-TphAB after 455 nm excitation (**B**).

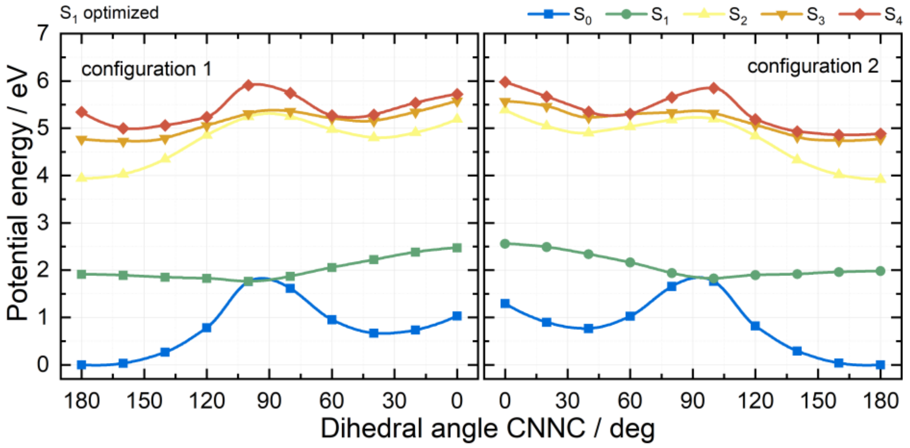

Figure S7. Potential energy curves for the torsion around one of the N═N double bonds in TphAB calculated for the S_1 optimized geometry at fixed values of the CNNC dihedral angle. Model 1 (TphAB-1) and model 2 (TphAB-2) correspond to the stable ground state geometries 1 and 2 (see figures above)

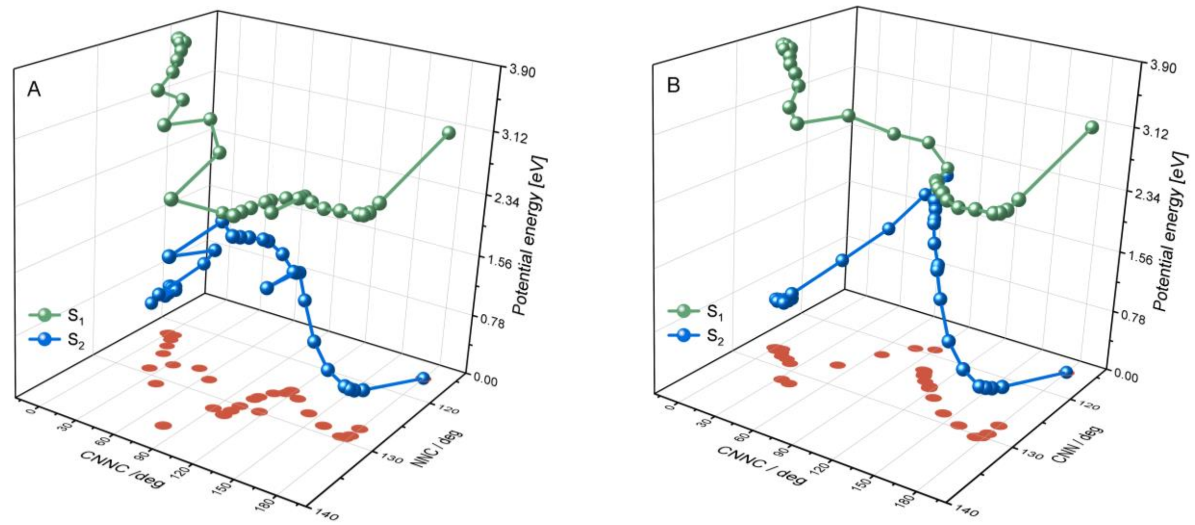

Figure S8. Potential energy curves obtained from the unconstrained/partially constrained optimization of (*E*)-TphAB-1 started from the Franck-Condon region on S_1. Note: NNC is the angle on the benzene side (**A**), while CNN is the angle on the thiophene side of the molecule (**B**).

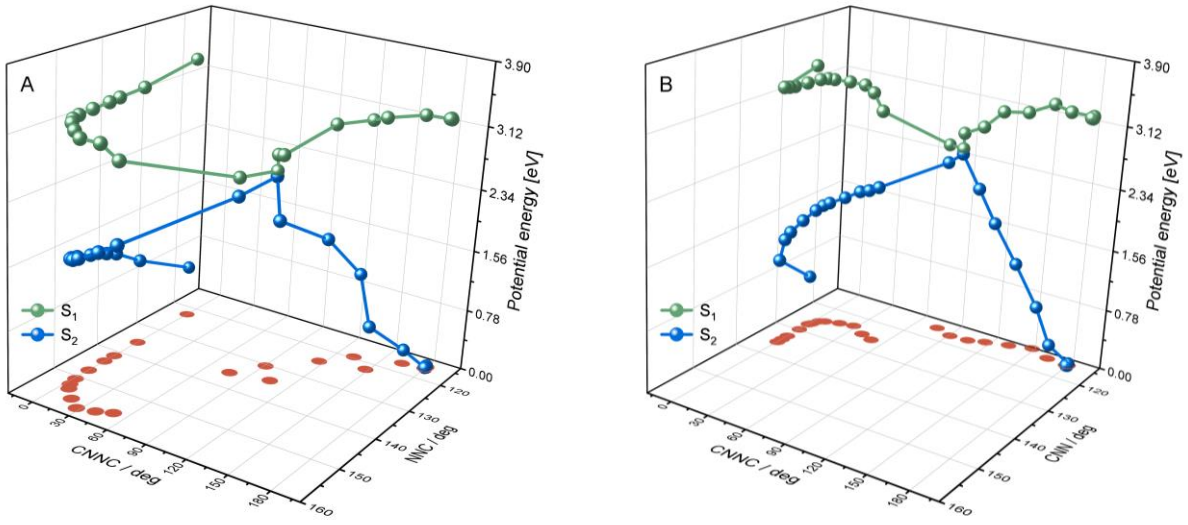

Figure S9. Potential energy curves obtained from the unconstrained optimization of (*Z*)-TphAB-1 started from the Franck-Condon region on S_1. Note: NNC is the angle on the benzene side (**A**), while CNN is the angle on the thiophene side of the molecule (**B**).
